# Discordant Responses to MAPK Pathway Stimulation Include Axonal Growths in Adult *Drosophila* Photoreceptors

**DOI:** 10.3389/fnmol.2018.00441

**Published:** 2018-12-04

**Authors:** Kirk L. Mecklenburg, Forrest P. Weghorst, Stephanie A. Freed, Joseph E. O’Tousa

**Affiliations:** ^1^Department of Biology, Indiana University South Bend, South Bend, IN, United States; ^2^Department of Biological Sciences, Eck Institute for Global Health, University of Notre Dame, Notre Dame, IN, United States

**Keywords:** dual leucine kinase, *Drosophila*, photoreceptors, degeneration, axon regeneration

## Abstract

Wallenda (WND) is the *Drosophila* member of a conserved family of dual leucine-zipper kinases (DLK) active in both neuronal regeneration and degeneration. We examined the role of WND over-expression on sensory neuron morphology by driving WND in multiple subtypes of *Drosophila* photoreceptors. WND overexpression under control of the pan-retinal GAL4 driver GMR causes multiple photoreceptor defects including cell death, rhabdomere degeneration, and axonal sprouting. Individual photoreceptor subtypes were assayed using GAL4 drivers specific for each photoreceptor class. Many R7 and R8 cells exhibit axonal sprouting while some show cell degeneration. Delaying the onset of WND overexpression until 20 days of age showed that older adult R7 cells retain the ability to initiate new axon growth. R1–6 photoreceptor cells degenerate in response to WND expression and exhibit rhodopsin loss and rhabdomere degeneration. RNAi knockdown of the MAPK signaling components Kayak (KAY) and Hemipterous (HEP) attenuates the WND-induced loss of Rh1 rhodopsin. UAS-induced HEP expression is similar to WND expression, causing degeneration in R1–6 photoreceptors and axonal sprouting in R7 photoreceptors. These results demonstrate that WND in adult *Drosophila* photoreceptor cells acts through MAPK signaling activity with both regenerative and degenerative responses. These photoreceptors provide a tractable experimental model to reveal cellular mechanisms driving contradictory WND signaling responses.

## Introduction

Neurons typically extend axonal projections during developmental stages until the growth cone reaches a target site and matures into a stable presynaptic bouton. However, in response to axonal damage, many neurons produce new axonal extensions and growth cones (Chisholm et al., 2016; He and Jin, 2016; Blanquie and Bradke, 2018). Other neurons, notably those within the CNS, fail to execute a regenerative response and instead commit to cell death. A large body of literature implicates the activity of a specific MAP3K known as the dual leucine-zipper kinase (DLK) in both axonal regrowth and neuronal degeneration processes (Welsbie et al., 2013; Lu et al., 2014; Chen et al., 2016; He and Jin, 2016). Following injury, a local rise in cAMP levels leads to PKA activation that in turns phosphorylates and activates DLK (Hao and Collins, 2017). A current research focus is the identification of the downstream effectors and associated mechanisms responsible for regenerative and degenerative outcomes following DLK activation.

*Drosophila* has proven to be a valuable genetic model to investigate the roles of DLK activity, both during development and following axonal injury. The* Drosophila* ortholog of DLK is coded by the *wallenda* (WND) gene. Enhanced WND activity triggers synaptic overgrowth in developing larval motor and sensory neurons (Collins et al., 2006; Wang et al., 2013) and axonal targeting defects in developing mushroom bodies (Shin and DiAntonio, 2011). WND overexpression in the developing visual system induces cell death and axon targeting defects (Ma et al., 2015; Feoktistov and Herman, 2016). WND activity also regulates neuronal responses to injury (reviewed in Brace and DiAntonio, 2017) and is required for cell degeneration when the axons of olfactory neurons are severed (Miller et al., 2009). Conversely, in motoneurons, WND function is necessary for regenerative sprouting following axonal damage (Xiong et al., 2010). Retrograde signaling from the site of injury to the cell body initiates a transcriptional program that is essential for the regenerative response (Horiuchi et al., 2007; Xiong et al., 2010; Rishal and Fainzilber, 2014). Thus, both degenerative and regenerative responses to WND signaling have also been described in the *Drosophila* system. The processes responsible for neuronal responses to injuries appear to be well conserved across vertebrates and invertebrates (Tedeschi and Bradke, 2013).

The adult *Drosophila* retina is composed of 800 simple eye units containing a small number of different photoreceptor sensory cell types. This experimental model has been exploited to gain a detailed understanding of the developmental processes controlling cell specification and differentiation (Treisman, 2013). To determine if this experimental model could also be used to study the WND-induced cellular responses to axonal injury, we evaluated the effects of WND overexpression in each of the photoreceptor cell types of the adult *Drosophila* visual system. Most R7 and R8 photoreceptors respond by inducing new axonal outgrowths. This regenerative capability is maintained in older adult animals. On the other hand, R1–6 photoreceptors and some of the R7/R8 cells show rapid cell degeneration. Genetic manipulation of the WND effectors Hemipterous (HEP) and Kayak (KAY) shows that both regenerative and degenerative outcomes of the WND response are mediated by canonical MAPK signaling pathways. Our study documents the usefulness of *Drosophila* photoreceptors for characterization of the cellular events leading to both regenerative and degenerative neuronal responses.

## Materials and Methods

### *Drosophila* Strains

*Drosophila* strains were obtained from other research laboratories or the Bloomington *Drosophila* Stock Center. Both male and female animals were analyzed. All animals used in the study were white eyed. The pigmentation contributed by the mini-white marker on many transgenes was routinely eliminated by placing the eye color mutations *cn* and* bw* into the analyzed genotypes. The drivers for the UAS/GAL4 system were GMR-GAL4 (P{w[+mC] = GAL4-ninaE.GMR}12), Rh1-Gal4 (P{ry[+t7.2] = rh1-GAL4}2), Rh3-GAL4 (P{w[+mC] = Rh3-GAL4}2), and Rh6-GAL4 (P{w[+mC] = Rh6-GAL4.D}3). The UAS strains were UAS Actin-GFP ({w[+mC] = UASp-Act5C.T:GFP}1), UAS-tdTom (P{w[+mC] = UAS-tdTom.S}2 and P{w[+mC] = UAS-tdTom.S}3, UAS-WND (P{w[+mC] = UAS-wnd.C}2), UAS-HEP (P{w[+mC] = UAS-hep.CA}4), and UAS-(RPR) (P{w[+mC] = UAS-rpr.C}14). The Rh3-LexA and LexAop CD2-GFP (Mazzoni et al., 2008) transgenes were used to label R7 axon boutons without using the UAS/GAL4 system. The tubGAL80^ts^ (P{w[+mC] = tubP-GAL80^ts^}2) strain was used to gain temperature control of UAS-WND expression.

### Histology

We removed heads and fixed overnight at 4°C in 4% paraformaldehyde, 5% sucrose prepared in phosphate buffered saline (PBS). The heads were rinsed in 5% sucrose/PBS, and treated overnight at 4°C in 5% sucrose/PBS. This solution was removed and replaced with 30% sucrose/PBS and samples treated overnight at 4°C. The 30% solution was removed and replaced with a 1:1 mix of 30% sucrose and O.C.T. Compound (OCT, Tissue-Tek) and heads treated for 4 h–4 days at 4°C. Heads were then placed in pure OCT and frozen onto the microtome chuck at −26°C. A Microm HM525 cryostat was used to produce 16 um ribbon sections of the head tissue. The tissue sections were collected on glass slides, dried, immersed in Vectashield mounting media (Vector Laboratories), and sealed with conventional microscope coverslips.

Brightfield images were collected on a Leica M205C dissecting scope equipped with a Leica DFC425 camera, LAS V4.0, and images were processed with the montage function. Confocal microscopy was performed on the Nikon A1R-MP Laser Scanning Microscope. All confocal images are maximum projection images generated using the Nikon NIS software package from adjacent optical section representing 8 μm–10 μm of tissue. Further manipulation of images (cropping, uniform adjustment of contrast and brightness) used Adobe Photoshop CC software. For all experiments, at least three heads of each genotype were examined for retinal cell body and axonal phenotypes, including the presence of axonal outgrowths at the synaptic regions in WND animals. The reported phenotypes were fully penetrant, being present in all experimental and none of the control samples.

### Protein Blots

Three to five flies of appropriate genotype were decapitated and heads solubilized in 5 μl/head solubilization buffer (Mecklenburg et al., 2015). Homogenates were spun briefly in a microcentrifuge, incubated at 42°C for 1 h, and 5 μl loaded onto a 4%–12% Nu-PAGE Bis-Tris gel (Invitrogen) in MOPS running buffer (Invitrogen) for gel electrophoresis. Proteins were then transferred to Immobilon-FL PVDF transfer membrane and incubated in Odyssey Blocking Buffer (Li-Cor) for 1 h. Labeling and wash reagents were as specified by Li-Cor IRDye protocols. Polyclonal antibodies against Rh1 rhodopsin, a reagent made previously in the lab, and monoclonal antibodies against Actin 5C, obtained from the Developmental Studies Hybridoma Bank at the University of Iowa, were applied overnight at 1:2,000 and 1:20,000 respectively. Detection of rhodopsin and Actin was carried out simultaneously by incubating the membrane for 30 min in IR Dye 680RD Goat Anti-mouse IgG and IR Dye 800CW Goat anti-rabbit IgG secondary antibodies (Li-Cor). Following rinses, the membrane was imaged on the Odyssey Infrared Imaging System (Li-Cor). For display of protein blot images, the data from the two infrared channels of the phosphoimager were converted to gray scale images using Adobe Photoshop CS software.

## Results

### Retinal Expression of WND Induces Photoreceptor Axon Sprouts

**Figure 1:**
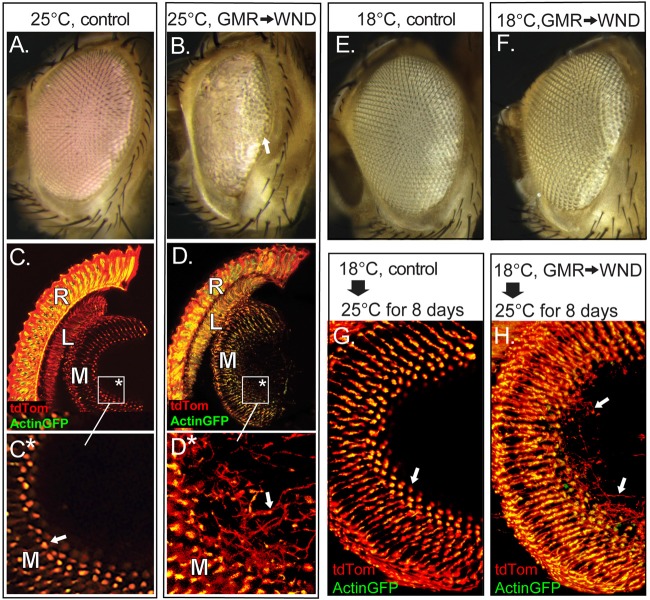
Wallenda (WND) expression in the adult retina causes axonal sprouting. **(A,B)** Flies carrying GMR-GAL4, tubGAL80^ts^ and reared at 25^o^C show normal external eye structure (**A**). Inclusion of UAS-WND within this genotype (**B**) resulted in a severe roughening of the external eye, with lens facets present only at the posterior edge (arrow). **(C–D)** The internal retinal structures of these flies at 8 days of age were visualized by inclusion of Actin-GFP and tdTom transgenes under GMR-GAL4 control. Confocal microscopy of cryosectioned heads of control flies (**C**, boxed region enlarged in C*) showed Actin-GFP and tdTom are detected within the retina (R) and in the first optic lobe, the lamina (L), and the second optic lobe, the medulla (M). Panel (C*) shows axons terminate at well-defined terminal buds (arrow). The inclusion of UAS-WND induces axonal outgrowth (sprouts) extending beyond the terminal bud of the photoreceptor axons within the medulla (**D**, boxed region enlarged in D*). The outgrowths are marked by an arrow in (D*). The retinal cell bodies are also disorganized in UAS-WND compared to controls. The individual rhabdomeres of control retina (R, **C**) are labeled by Actin GFP, while individual rhabdomeres appear more diffused in WND retina (R, **D**). **(E,F)** Flies carrying GMR-GAL4, tubGAL80^ts^, and reared at 18°C show the wild type external eye morphology (**E**). Inclusion of UAS-WND in this genotype does not alter the external eye morphology when flies are reared at 18°C (**F**). **(G,H)** Control flies reared at 18°C until eclosion then shifted to 25°C for 8 days show the typical pattern and structure of axon terminals within the medulla (arrow, **G**). The presence of UAS-WND in this genotype results in axon sprouts within the medulla (arrows, **H**).

Previous work showed that WND expression during eye development induced the formation of a small eye phenotype due to extensive death in third instar eye disc cells posterior to the morphogenetic furrow (Ma et al., 2015). That study made use of the driver GMR-GAL4, expressed in the larval eye-antenna imaginal disk immediately following the progression of the morphogenetic furrow and prior to the determination and differentiation of both neurons and retinal support cells (Freeman, 1996). We used the constitutively expressed temperature sensitive GAL4 suppressor tubGAL80^ts^ to investigate if suppression of this detrimental effect of WND expression during early development would induce a regenerative WND effect in adult photoreceptors. Control flies carrying GMR-GAL4 and tubGAL80^ts^ but reared at 25°C to curtail tubGAL80^ts^ activity show a normal eye morphology (Figure 1A), but introduction of the UAS-WND element into this genotype produced a small rough eye phenotype (Figure 1B) as expected from GMR-triggered WND expression (Ma et al., 2015). The WND-induced rough eye was not uniform, notably showing a greater disorganization in the anterior regions relative to posterior regions (arrow).

To examine internal retinal structures, two fluorescent reporters were included in these genotypes. These reporters were tdTomato (tdTom), a diffusible protein labeling the cytoplasm, and Actin-GFP, labeling the actin-rich rhabdomeres of the photoreceptors. Vertical tissue sections through the retina of control flies (Figures 1C,C**) shows localization of Actin-GFP to the photoreceptor rhabdomeres, and tdTom throughout the photoreceptor cell body as well as photoreceptor axonal projections into the lamina (L) and within the terminal buds located in the medulla (M). In contrast, the retina of WND-expressing flies show disorganization of actin localization within photoreceptor cell bodies. These animals also showed evidence of axonal sprouts within the medullar synaptic region (arrow, Figures 1D,D**). For this and all subsequent histological analyses, three or more animals were examined for each genotype to confirm the full penetrance of all reported phenotypes.

To distinguish the WND effect on the developing retina from the WND effect on the adult retina, we raised flies at 18°C to allow tubGAL80^ts^ suppression of GMR-GAL4 induced WND expression. These flies exhibited an external eye morphology similar to control flies (Figures 1E,F) These results indicate that limiting WND activity during retinal developmental stages prevented the small, rough eye phenotype that results when the same animals were reared through development at 25°C (Figure 1B).

To examine the effect of increased WND activity exclusively in the adult retina, we raised the control (lacking UAS-WND) and experimental (UAS-WND) animals through eclosion at 18°C, then shifted the flies to 25°C for 8 days. Both control and experimental animals possessed normal external eye morphology. Histological examination of these flies showed that WND expression resulted in axonal outgrowths within the medulla (arrows, Figure 1H) that was not observed in controls (Figure 1G). These results indicated that the small rough eye phenotype results from increased WND activity during early retinal development, while the axonal sprouting phenotype is due to WND overexpression at later time points.

### WND Induces Axon Sprouting in R7 and R8 Cells

**Figure 2:**
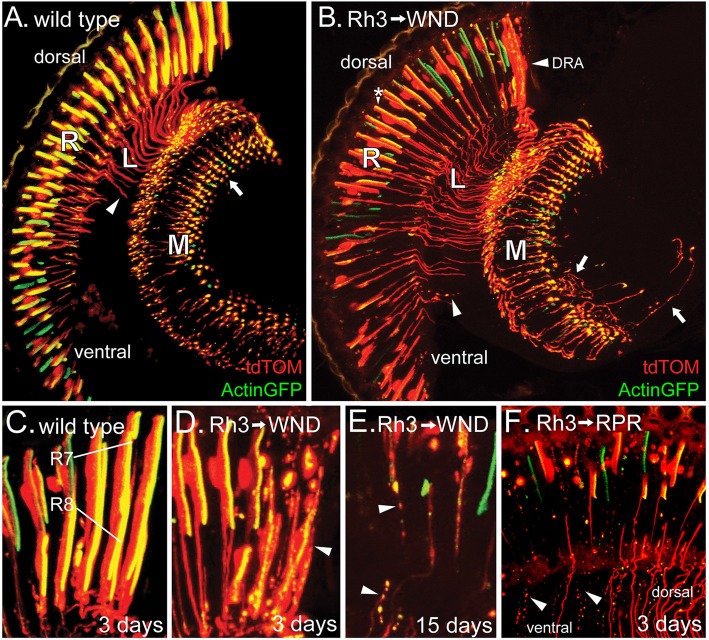
WND expression in adult R7 photoreceptors triggers both axon sprouting and cell degeneration. **(A)** In 3-day old animals expressing Actin-GFP and tdTom under Rh3 rhodopsin control, Actin-GFP is localized to the rhabdomeres of the R7 cell bodies of the retina (R) as well as the axon terminals (arrow) within the medulla (M). The tdTom protein is not membrane-associated and so localized to the cell bodies, axon terminals, and the axon tracts (arrowhead) traversing the lamina (L). **(B)** When WND is expressed along with Actin-GFP and tdTom under Rh3 rhodopsin control, the Actin-GFP labeled rhabdomeres are reduced in size (*), some axon tracts through the lamina (L) are atrophied into bead-like remnants (arrowhead), and many axon termini have extended axonal sprouts (arrows). The dorsal rim area (DRA) of the retina is most affected by WND expression. **(C–F)** Within the DRA, both R7 and R8 cells express Rh3. Control flies **(C)**, carrying Actin-GFP and tdTom show the expected localization of these proteins. In 3-day old flies expressing WND, the R7 and R8 cells within the dorsal rim **(D)**, lack the integrity of the Actin-GFP rhabdomeric structures and tdTom labeled cell bodies (arrowhead). At older ages **(E)**, these R7 and R8 cells show additional deterioration, producing tracks of cellular debris containing both Actin-GFP and tdTom proteins (arrowheads). Regulatory protein reaper (RPR) expression in R7 cells **(F)** produces similar tracks of bead-like debris (arrowheads) in 3-day old flies. The remaining axons are largely those of R7 cells located in the dorsal region. Axons and axon debris tracks are absent from a large portion of the ventral region.

The GMR-GAL4; UAS-WND genotype induces increased WND expression in retinal precursor cells following the sweep of the morphogenetic furrow (Freeman, 1996) that is completed by 10% pupal development (Wolff and Ready, 1993). To examine WND effects later in development, after determination and differentiation of photoreceptor cells, we drove WND expression with rhodopsin promoter-GAL4 drivers. All rhodopsin promoters become active at about 80% pupal development (Kumar and Ready, 1995; Earl and Britt, 2006). Rh3 rhodopsin is expressed in 70% of R7 photoreceptors and the R8 photoreceptors of the dorsal rim area (DRA; Fortini and Rubin, 1990). The cell body, axons and synaptic regions of these photoreceptors were visualized by expressing tdTom and Actin-GFP under control of the Rh3 promoter (Figure 2A). When WND was also expressed, these cells possessed a large number of axonal extensions near the synaptic regions (arrows, Figure 2B).

The R7 photoreceptor cells of different regions show non-uniform responses to WND expression. Within the ventral region, the photoreceptor cells often lose the integrity of rhabdomeres (assessed by the Actin-GFP labeling in Figure 2B). The axon projections within the lamina and medulla are often broken up into small bead-like structures (arrowhead, Figure 2B). The photoreceptor cells of the DRA (Figure 2B, labeled) show the most pronounced degenerative response. Figure 2C displays the DRA of wild type. Both R7 and R8 photoreceptors are labeled because within the DRA, the Rh3 promoter is active in both cell types. Figure 2D shows a similar view of the DRA in the presence of UAS-WND. WND expression induces clear signs of degeneration at this 3-day time point. The R7 and R8 cells show further degeneration at older ages, notably being broken up and dispersed into smaller structures resembling a track of apoptotic bodies (arrowheads, Figure 2E). To determine if induced apoptosis would generate a similar photoreceptor phenotype, we expressed the known apoptosis inducer Reaper (regulatory protein reaper, RPR) in the R7 photoreceptors. Analysis of 3-day old animals showed that RPR is effective in inducing R7 cell death (Figure 2F). The tracks of apoptotic bodies produced by RPR (arrowhead) are morphologically similar to the debris tracks induced by WND expression. The absence of axons and axon debris tracks in a large part of the ventral region suggests that the remnants of axons have been cleared by this 3-day time point.

**Figure 3:**
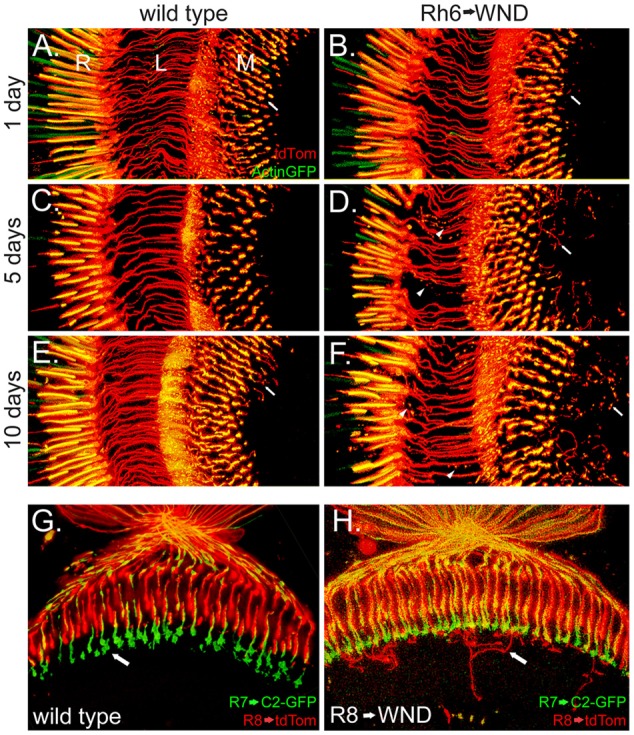
Induction of axonal sprouts by WND expression in R8 photoreceptors. **(A–F)** The Rh6 promoter was used to assay the effect of WND expression in R8 photoreceptors. The R8 cells are marked with Actin-GFP and tdTom. Similar vertical sections are shown in each micrograph, as labeled in panel (**A**; proximal retina (R), the lamina (L) and medulla (M)). Three different ages are shown. In the absence of Rh6→WND expression (left panels), axon structure is consistent at all ages, and axons terminate in the medulla (arrow, **A**). Limited axon sprouts are seen at 10 days (arrow, **E**). With Rh6→WND expression (right panels), axon sprouts are detectable at the 1-day time point and are more significant at the older time points (arrows, **B,D,F**). In addition, atrophy of some axons is evident at the 5-and 10-day time points (arrowheads, **D,F**). **(G,H)** Horizontal sections with the R7 axon terminals (arrow, **G**) marked with C2-GFP and R8 cells with tdTom in 5-day old control (**G**) and Rh6→WND (**H**). The axon sprouts caused by WND expression in the R8 cells extend into neuronal tissue beyond the R7 axon terminals and then grow laterally within deeper layers of the medulla (arrow, **H**).

The Rh6 promoter (Tahayato et al., 2003) was used to determine if increased WND expression would initiate axonal outgrowths in R8 cells. Results from this approach are shown in Figure 3. Control animals retained the expected axon structure at all ages, with the caveat that some small axon sprouts were observed in the wild-type R8 at 10 days post-eclosion (Figures 3A,C,E). Rh6-driven WND expression induced axon sprouts at earlier time points and increased both the number and length of sprouts at the later time points (arrows, Figures 3B,D,F). Some axons, however, have disintegrated into small blebs at the later time points (arrowheads, Figures 3D,F).

The medulla is organized such that R7 and R8 photoreceptors form synapses at different layers. R8 photoreceptors form synapses in the M3 layer while R7 forms synapses in the deeper M6 layer (Fischbach and Dittrich, 1989). We investigated the possibility that the more proximal position of M3 and layout of cell types within the medulla might influence the outgrowth and targeting of the R8 axon sprouts. We marked R7 cell boutons within the M6 layer by using Rh3LexA to drive expression of the membrane marker CD2-GFP. This analysis revealed that WND-induced R8 axon sprouts frequently extended beyond the M6 layer, then turn and grow laterally within one of the deeper layers (Figure 3H). These observations suggest that the R7 synaptic region is unlikely to contain cues influencing the R8 axon outgrowths, but lateral growth cues may be present in more proximal layers of the medulla.

### WND Expression Induces Axon Sprouting in Older Flies

**Figure 4:**
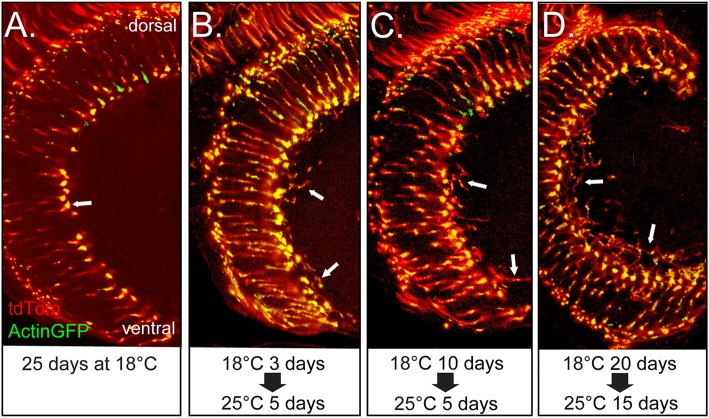
Adult R7 photoreceptor cells retain the ability to initiate axon sprouts at older ages. All panels show views of the medulla for the same genotype in which the R7 cells express GAL4 and tubGAL80^ts^ and contain UAS-WND, UAS-Actin-GFP, and UAS-tdTOM. For this genotype, the levels of WND, Actin-GFP, and tdTom are dependent on GAL4 activity. The GAL4 activity is suppressed by tubGAL80^ts^ at lower temperatures. The animals were reared under different temperature regimes as designated under each of the micrographs. **(A)** Flies reared at 18°C for 25 days show typical axon terminal structure lacking evidence of axon sprouts (arrow). **(B–D)** Flies reared at 18°C for 3, 10 and 20 days post-eclosion, then shifted to 25°C, for 5 or 15 days show axon sprouts (arrows in all panels).

We considered the possibility that WND’s induction of axonal sprouts might be restricted to maturing photoreceptors of the late pupal development stage. To assess the response to WND expression initiated later in the adult, we introduced tubGAL80^ts^ into the Rh3-GAL4, UAS-WND genotype. No axonal sprouts were observed when tubGAL80^ts^ adults were placed at 18°C for 25 days (Figure 4A). These results are consistent with the expectation that at this temperature tubGAL80^ts^ limits GAL4 activity and thus expression of WND. In contrast, axonal sprouting was observed in flies shifted from 18°C to 25°C at different ages then reared at 25°C for 5 days or 15 days (arrows, Figures 4B–D). Control genotypes lacking the UAS-WND construct subjected to these experimental regimes showed no axonal sprouting. These results establish that the R7 photoreceptors retain the ability to initiate axon growths in response to WND signaling during their adult life.

### Photoreceptor Cells R1–6 Degenerate in Response to WND Expression

**Figure 5:**
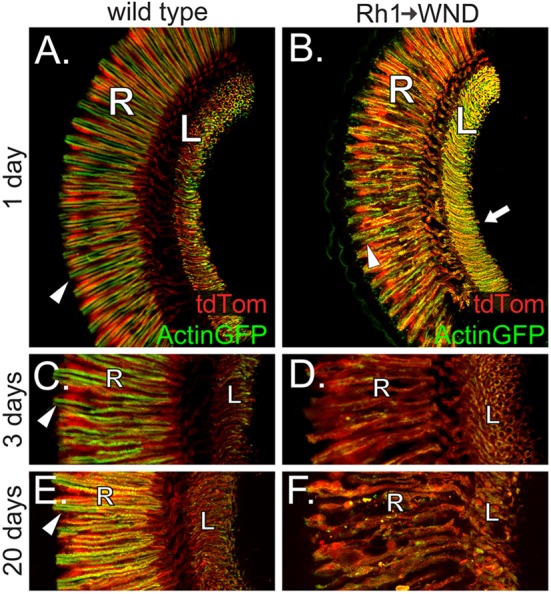
WND expression in R1–6 photoreceptors causes rhabdomere collapse and cell degeneration. **(A)** Confocal image shows that R1–6 photoreceptor cells of wild type flies retain Actin-GFP labeling principally within rhabdomeric membranes (arrowhead) and the axon terminals within the lamina (L). The retinal layer (R) is also labeled. tdTom is distributed throughout the photoreceptor cells. **(B)** At 1 day post-eclosion, the R1–6 cells of flies expressing WND under Rh1 rhodopsin control show redistribution of Actin-GFP. Compared to controls, the rhabdomeres are disheveled (arrowhead), and the axons traversing from the retina to the lamina are bloated. The axon terminal regions within the lamina (arrow) appear enriched for Actin-GFP, but axon sprouts are not detectable. tdTom distribution is similar to wild type. **(C,D)** At the 3-day time point, Actin-GFP is principally localized to the rhabdomeres (arrowhead) of the R1–6 photoreceptors in wild type (**C**). Lower levels of Actin-GFP are found at the axon terminals within the lamina (L). tdTom distribution throughout the photoreceptors allows demarcation of the retina and lamina regions. WND expression has caused extensive R1–6 cell degeneration by 3 days, including loss of rhabdomere structure and general disintegration of retinal cell bodies, axons, and synaptic terminal regions. The retinal layer (R) and lamina (L) are labeled. **(E,F)** At 20 days, Actin-GFP is principally localized to the rhabdomeres (panel E, arrowhead) of the R1–6 photoreceptors in wild type. Some WND-expressing cells possess an amorphous structure yet are intact and capable of Actin-GFP and tdTom expression. The retinal layer (R) and lamina (L) are labeled.

The largest class of photoreceptors consists of the R1–6 cells expressing Rh1 rhodopsin. The UAS-WND transgene was driven by Rh1-GAL4 to assess the effect of WND expression in adult R1–R6 cells. Actin-GFP and tdTom expression were again used to reveal the basic aspects of the R1–6 cell organization (Figure 5). WND expression causes loss of rhabdomere integrity at the 1-day time point (arrowheads, Figures 5A,B). By 3 days, WND expression has caused the extensive degeneration of the R1–6 cells (Figures 5C,D), yet tdTom expression is still present. Analysis of retinas at 3 days shows that WND-induced degeneration proceeds rapidly (compare Figure 5B vs. Figure 5D). At 20 days, WND expression has caused the extensive degeneration of the R1–6 cells relative to the wild-type (Figure 5F vs. Figure 5E). The presence of tdTom in the 20-day WND retina shows some R1–6 cells persist. Unlike the R7 and R8 cells, there is no evidence of axonal sprouting within the synaptic regions of the R1–6 cells at any time point. However, we cannot exclude the possibility of small axonal outgrowths because these could not be identified given the high density of R1–6 termini in the lamina.

**Figure 6:**
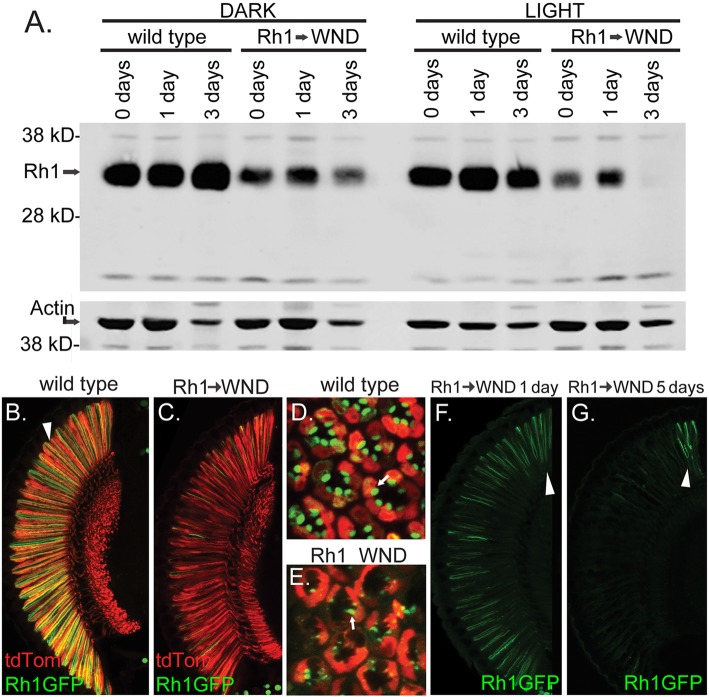
WND-induced loss of rhodopsin in R1–6 photoreceptors. **(A)** Protein blot results showing Rh1 rhodopsin levels in controls and in experimental flies expressing WND in the R1–6 photoreceptors. Proteins were extracted from heads within 4 h of eclosion (0 days), at 1 day, and at 3 days after rearing in darkness (left) or under 24 h light (right). The lower image shows the detection of Actin on the same membrane as a loading control. **(B–E)** Confocal image of retinas showing Rh1-GFP localization in control and with WND expression. Rh1-GFP is localized to rhabdomeres of control flies (arrowhead, **B** and arrow, **D**). WND expression depletes rhodopsin content. The residual rhodopsin remains localized to the degraded rhabdomeres (arrow, **E**). The soluble tdTom protein is found within the cytoplasmic region of the photoreceptors. **(F,G)** Rhodopsin localization at the 1-and 5-day time points in the presence of WND expression. Rhodopsin content is retained only within the DRA (arrowheads) at the 5-day time point.

We probed the expression and cellular location of the Rh1 rhodopsin to further investigate the effect of WND expression on the R1–6 cells. Figure 6A shows the result of a protein blot examining Rh1 rhodopsin content in control and WND animals at the 0, 1 and 3-day time points. In controls reared under dark conditions (left), flies at eclosion (0 days) possess high levels of Rh1 rhodopsin. Rhodopsin content is reduced in the experimental WND animals relative to the control animals at all ages. Rhodopsin content is lowest at the 3-day time point, likely due to cell deterioration triggered by WND expression. Rearing flies in light conditions accelerated the WND-induced loss of rhodopsin (Figure 6A, right) such that rhodopsin is not detectable at 3 days.

The cellular distribution of the Rh1 rhodopsin was investigated by use of a UAS Rh1-GFP transgene (Pichaud and Desplan, 2001). In control flies, Rh1-GFP shows the expected Rh1 localization within the rhabdomeres of the R1–6 cells when viewed both by vertical (arrowhead, Figure 6B) and retinal cross sections (Figure 6D). tdTom marks the cytoplasmic compartment of these cells. In Rh1 cells expressing WND, the rhodopsin content is markedly reduced (Figure 6C). The residual rhodopsin continues to be localized within the degenerating rhabdomere structures of these cells (arrow, Figure 6E). The loss of Rh1-GFP rhodopsin is not uniform across the retina. Figure 6F isolates the Rh1-GFP expression for the 1-day WND retinal section displayed in Figures 6C,G shows a corresponding view of a 5-day WND retina. At the 1 day timepoint, the DRA appears enriched for rhodopsin content (arrowhead, Figure 6F). By the 5-day timepoint, the Rh1-GFP content is largely restricted to the photoreceptors of the DRA (arrowhead, Figure 6G).

### WND Acts on R1–6 and R7 Cells Through the MAPK Kinase Hemipterous

**Figure 7:**
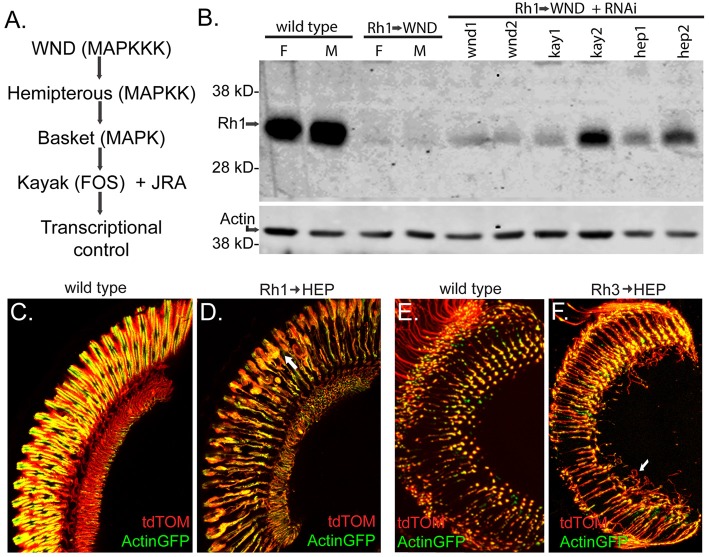
The MAP Kinase pathway downstream of WND is involved in R1–6 rhodopsin loss and cell degeneration, and in R7 cell axonal sprouting. **(A)** The MAP Kinase pathway shows *Drosophila* gene names with common MAPK pathway names in parentheses. WND is specified as the MAPKKK in this diagram; four other *Drosophila* MAPKKK members signal through this pathway (Stronach et al., 2014). **(B)** Protein blot analysis shows the effect of RNAi treatments on WND-induced loss of Rh1 rhodopsin. Controls at left show high levels of Rh1 protein in female (F) and male (M) at 3 days, while low rhodopsin content is present when WND is expressed in these cells. The lanes to the right show better retention of rhodopsin when UAS-RNAi constructs targeting the indicated genes are expressed simultaneously with WND. These strains are wnd1:P{TRiP.JF02675}attP2, wnd2:P{TRiP.GL00282}attP2, kay1:P{TRiP.JF01273}attP2, kay2:P{TRiP.HMS00254}attP2, hep1:P{TRiP.JF03137}attP2, and hep2:P{TRiP.GL00089}attP2. The lower image shows the detection of Actin as a loading control. **(C,D)** Images of the retina shows the effect of UAS HEP expression on R1–6 photoreceptors. At 3 days, controls retain normal retinal structure whereas HEP expression causes R1–6 cell degeneration (arrow). The cell markers tdTom and Actin-GFP were used as previously described. **(E,F)** Sections through the medulla shows the effect of HEP expression on the axon terminal of R7 photoreceptors. At 3 days, controls retain normal axonal terminals, whereas HEP expression causes axon sprouting (arrow).

In the canonical WND-triggered cellular signaling pathway, WND, a MAPK kinase kinase, phosphorylates the MAPK kinase Hemipterous (HEP), which in turn activates a c-Jun N-terminal kinase (JNK), encoded by the gene *basket* (*bsk*). BSK then triggers activation of the transcription factors FOS, encoded by the gene *kayak* (*kay*), and Jun-related antigen (JRA; Stronach, 2005). This cascade is summarized in Figure 7A. We sought evidence that WND acted through this pathway in photoreceptors to trigger rhabdomere degeneration and axonal sprouting. We first examined WND’s effect on Rh1-GFP rhodopsin levels in the presence of UAS-RNAi transgenes (Perkins et al., 2015) against *wnd*, *kay* and *hep*. One transgenic RNAi line targeting *kay* and one transgenic RNAi line* hep* markedly improved the Rh1-GFP fluorescence levels in the intact eye. This result prompted us to evaluate native Rh1 levels in these genotypes. Figure 7B shows these results for 3-day old flies. All RNAi elements targeting *wnd, kay and hep* showed some increase in Rh1 content. The two RNAi elements (kay2 and hep2) identified by direct evaluation of Rh1-GFP showed the largest increase in Rh1 content. Thus, these results are consistent with the hypothesis that HEP and KAY are required downstream of WND to reduce Rh1 levels.

We reasoned that the photoreceptor response to elevated HEP expression might be similar to that seen for elevated WND expression if this MAPK signaling pathway triggered the response. To test this possibility, we increased HEP expression in R1–6 photoreceptors by placing a UAS-HEP element under control of Rh1-GAL4. These flies exhibited R1–6 rhabdomere degeneration and the other morphological phenotypes described previously for WND (Figure 7D, compare to control in Figure 7C). We also placed the same UAS-HEP element under Rh3-GAL4 and showed that R7 photoreceptors respond to HEP expression by producing long axonal sprouts within the medulla (Figure 7F, compare to control in Figure 7E). Together these results demonstrate that WND activation of the MAP kinase pathway is responsible for both the regenerative and degenerative effects of WND expression in photoreceptors.

## Discussion

The *Drosophila* WND protein is a conserved dual leucine kinase involved in the initiation of both regenerative and degenerative responses in a variety of neuronal cell types. We demonstrate here that *Drosophila* photoreceptors are a tractable experimental system to examine both of these responses. An initial study showed that WND expression within the developing *Drosophila* retina (Ma et al., 2015) caused pervasive cell death resulting in a small and disorganized eye. These studies made use of GMR-driven WND expression providing pan-retinal expression early in retinal development. Using this same GMR expression scheme, we documented a second and distinct consequence of WND expression. This is that the retinal axonal projections terminating in the second optic lobe (the medulla) possess outgrowths extending beyond the location of the synaptic terminal bud. These outgrowths suggested that retinal WND activity is capable of a regenerative response, triggering new axonal growth processes.

To further investigate this phenomenon, we restricted WND expression to specific photoreceptor cell classes by using rhodopsin promoters. The Rh1 rhodopsin is expressed in R1-R6 photoreceptor cells, the Rh3 rhodopsin in R7 cells, and the Rh6 rhodopsin in R8 cells. All rhodopsin promoters are active at about 80% pupal development (Kumar and Ready, 1995; Earl and Britt, 2006). Earlier, at 20% pupal development, the R1–6 photoreceptors have extended their axons into the lamina and completed the assembly of synaptic structures (Fischbach and Hiesinger, 2008). The R7 and R8 cell axons have extended into the medulla by this 20% time point, and they subsequently undergo a second stage of target selection that results in assembly of the adult configurations by 70% pupal development (Ting et al., 2005). Thus, rhodopsin promoters allowed initiation of WND expression subsequent to the developmental decisions specifying the identity of photoreceptor cell types and the completion of axonal targeting and synaptogenesis.

Both R7 and R8 photoreceptor axons terminate in the medulla and therefore are likely responsible for the axonal sprouts observed following GMR-driven WND expression. Consistent with this expectation, using the Rh3 promoter to drive WND expression in the R7 cells caused these photoreceptors to produce axonal spouts. However, this is not a uniform response, as extensive R7 cell degeneration is also observed. The most pronounced and rapid degeneration occurs in the R7 cells of the DRA. The ventral retinal region shows a slower rate of degeneration than the DRA, and the lowest rate is seen within the dorsal region. The differences in the levels of WND produced in each of these groups of R7 cells might explain these results. R7 cells in the DRA are specialized to allow polarized light detection, possessing larger rhabdomeres than elsewhere in the retina (Wernet et al., 2003). It is possible that Rh3 is expressed at higher levels in the DRA to support the specialized role. In contrast, many R7 cells within the dorsal region known as the dorsal third (Mazzoni et al., 2008) coexpress Rh3 and Rh4, thus producing Rh3 at a lower level than cells only expressing Rh3. In our experimental set up, the level of WND expression is dependent on the activity of the Rh3 promoter. This ranking: DRA highest, ventral intermediate, and dorsal region lowest, suggests that higher levels of WND expression will trigger more rapid degeneration. It is possible that individual cells initiate a regenerative response that is then negated by a subsequent degenerative response. We considered the possibility that only recently differentiated photoreceptor cells possess the capacity for a regenerative response. This is not the case, however, as axon sprouting is observed when WND expression is induced in R7 photoreceptors at 3, 10 and 20 days of adult age.

The R8 photoreceptors expressing Rh6 also produce axonal sprouts in response to WND expression. Unlike R7 cells, no regional differences in the R8 axonal response were observed. This may reflect that the Rh6 promoter, unlike the Rh3 promoter, has uniform activity in all retinal regions. Some R8 cells, however, do commit to a degenerative response. Fragmented axons are seen at both the 3-and 10-day time points. The fragmented axons present at these two time points likely result from commitment of different cells to degeneration, as fragmented axons produced by RPR expression are eliminated within 3 days. We have thus demonstrated that both R7 and R8 photoreceptors respond to WND activity by initiating conflicting activities, resulting in both axonal outgrowth and cell degeneration processes. These results are consistent with the view that an initial regenerative response can switch to a degenerative response. The discordant responses of axon sprouting and axon degradation in R7 and R8 may be mechanistically related to similar processes during axon growth and pruning where both progressive and regressive events are occurring (Riccomagno and Kolodkin, 2015). A clear demonstration of this, however, will require experimental designs incorporating the labeling of individual photoreceptor cells.

In contrast to the R7 and R8 photoreceptors, WND expression in R1–6 photoreceptors does not trigger major axonal sprouting, rather causing a uniform degenerative response. One regional distinction is recognized, namely that R1–6 cells within the DRA degenerate at a slower rate. As with R7 photoreceptors, this result is likely the result of R1–6 photoreceptor specializations within the DRA. In most of the retina, R1–6 photoreceptors at 1 day post-eclosion show rapid loss of rhodopsin and the microvillar structure of the rhabdomere. Many R1–6 cell bodies, however, persist past the 20-day time point. This contrasts with the behavior of R7 and R8 cells, which completely break down and their cellular fragments cleared within a few days following the commitment to degeneration. Thus, it is likely that there are unique features of the R1–6 cell WND response, not simply the lack of detectable regenerative axonal sprouting.

Many experimental paradigms have demonstrated that stimulation of WND activity by axonal damage is required for regenerative and degenerative neuronal responses (Brace and DiAntonio, 2017; Hao and Collins, 2017). We show here that the overexpression of WND in the absence of damage will trigger similar regenerative and degenerative responses in adult *Drosophila* photoreceptors. Our results are consistent with previous studies in neurons and other sensory cells showing WND overexpression is capable of initiating a damage response (Collins et al., 2006; Miller et al., 2009; Wang et al., 2013). With increased expression, the summation of WND basal activity must bring the total cellular WND activity above the response threshold. Positive feedback loops, such as those documented for vertebrate DLK (Huntwork-Rodriguez et al., 2013) and WND (Hao et al., 2016) may also play a role. Stimulation of WND activity by overexpression does not allow WND activation to be restricted to the site of axonal injury. Cytoskeletal remodeling occurring at the injury site likely contributes to the initiation of the axonal response (He and Jin, 2016). It is noteworthy that our study shows that in the absence of an injury site, the axonal sprouts occur within the synaptic region. The existing structure of the synapse, or its ability to remodel in response to WND signaling, may be key to regenerative capacity. It is also the case overexpression of WND by results in differences in the magnitude and duration of WND activity relative to injury-induced stimulation.

Our results show that WND activation alone triggers the processes responsible for both degenerative and regenerative outcomes in *Drosophila* photoreceptors. Both degenerative and regenerative outcomes of WND overexpression are mediated through the MAPK signaling pathway active in the neuronal injury response. We also demonstrate that axon growth can be reinitiated in the absence of axon damage even in older adults. This is a most tractable experimental model, capable of revealing signaling pathways and cellular mechanisms underlying both degenerative responses and regenerative capabilities of differentiated neurons in distinct cellular contexts.

## Author Contributions

KM, FW and JO’T designed the research. KM, FW, SF and JO’T performed the research and analyzed the data. KM, and JO’T wrote the article.

## Conflict of Interest Statement

The authors declare that the research was conducted in the absence of any commercial or financial relationships that could be construed as a potential conflict of interest.
